# First- and Third-Person Perspectives in Psychotic Disorders and Mood Disorders with Psychotic Features

**DOI:** 10.1155/2011/769136

**Published:** 2010-08-24

**Authors:** Lucrezia Islam, Silvio Scarone, Orsola Gambini

**Affiliations:** Psychiatric Branch, Department of Medicine, Dentistry and Surgery, University of Milan Medical School, San Paolo Hospital, Via Antonio di Rudinì 8, 20142 Milan, Italy

## Abstract

Lack of insight, very frequent in schizophrenia, can be considered a deficit in Theory of Mind (ToM) performances, and is also found in other psychiatric disorders. In this study, we used the first- to third-person shift to examine subjects with psychotic and psychotic mood disorders. 92 patients were evaluated with SANS and SAPS scales and asked to talk about their delusions. They were asked to state whether they thought what they said was believable for them and for the interviewer. Two weeks later, 79 patients listened to a tape where their delusion was reenacted by two actors and were asked the same two questions. Some patients gained insight when using third-person perspective. These patients had lower SAPS scores, a lower score on SAPS item on delusions, and significant improvement in their SAPS delusion score at the second interview. Better insight was not related to a specific diagnostic group.

## 1. Introduction

Patients suffering from schizophrenia are incapable of recognizing and monitoring the self or nonself induced character of their own thoughts. This monitoring capacity, which separates self-generated and world-generated perception, is very important to distinguish between imagination and normal perceptions. If this monitoring capacity is disturbed, self-induced perception is experienced as world-induced [1].

There is increased evidence that patients with schizophrenia have difficulties in social cognition which requires sophisticated judgements about other people's mental states. People with schizophrenia have worse performance profiles in tasks that require the interpretation of social inferences underlying indirect speech. 

Frith and collaborators, first applied the Theory of Mind deficit hypothesis to schizophrenic patients, and since then many studies have attempted to define the concept in a way that could be tested experimentally [2–4].

It has been suggested that some paranoid symptoms and behavioural signs could be a consequence of difficulties in making inferences about the intentions and beliefs of others [5]. Many clinical schizophrenic symptoms can be reinterpreted as a disturbance of the “self-monitoring capacity.” Impaired monitoring ability in schizophrenic patients can lead to serious problems in understanding subtle, context-dependent changes in the content and significance of communication [6].

Lack of insight is a common symptom in schizophrenia and can be considered a critical manifestation of impaired ToM abilities. Insight in schizophrenia is operationally defined according to five dimensions which include the patient's awareness of mental disorder, awareness of the social consequences of disorder, awareness of the need of treatment, awareness of symptoms, and attribution of symptoms to disorder [7, 8]. Following these criteria, the lack of insight can be considered an aspect of an impaired self-monitoring capacity. 

Lack of insight is not only found in schizophrenia, but can also be found in other psychotic disorders and in psychotic mood disorders [9–12]. Neurocognitive deficits have been described both in schizophrenia and mood disorders, and have been proposed to reflect underlying neurobiological dysfunction [13]. 

The lack of awareness of illness is not specific for psychiatric patients and can be found also in neurological patients. An example is emi-neglect syndrome, in which anosognosia for hemiplegia and lack of awareness of illness are fundamental symptoms. In their studies [14, 15], Tegner and Marcel asked anosognosic hemiplegic patients about their performances with their paralyzed limbs. The patients described their limbs as normal. 

However, if the authors asked the same patients, “If my arm was paralyzed, could I shuffle a pack of cards?” some of the patients responded “Of course not.” These results indicate that in some cases, the passage from a first-person to a third-person perspective can change the patient's awareness about his/her illness. We have previously hypothesized [16] that this phenomenon could be a particular aspect of a Theory of Mind task and have shown that 30 schizophrenic delusional inpatients modified their opinion about their delusions shifting from the first to the third person. 

In the current study, we have evaluated 92 patients with psychotic and psychotic mood disorders using first- to third-person shift. This task resembles the ToM second-order stories. Our hypothesis was that patients should gain insight and self-monitoring capacity when listening to their own delusions presented in a neutral context.

## 2. Subjects

92 (35 women, 57 men) patients participated in the study. Patients were recruited from the inpatient service of the Psychiatric Branch of the Department of Medicine, Surgery, and Dentistry of the University of Milan Medical School. All patients in the study had been admitted because of their acute psychotic state. Only those who volunteered and gave informed consent were included in the study. Informed consent with respect to the purposes and the procedure of the study was obtained from all subjects prior to starting the testing procedure. 

Diagnoses were made according to the DSM-IV-TR criteria by the authors (S. Scarone and O. Gambini). 69 subjects had a diagnosis of schizophrenia (21 undifferentiated, 48 paranoid), 7 had a diagnosis of psychotic mood disorder (6 of Bipolar Disorder, 1 of Delusional Depression), and 16 subjects had a diagnosis of other psychotic disorders (9 of Psychosis not otherwise specified, 2 of Schizoaffective disorder, and 5 of Delusional Disorders). Inclusion criteria were one of the previously cited diagnoses and the presence of delusions. Exclusion criteria were organic illnesses involving the central nervous system, current substance abuse and/or past and current alcohol dependence, and clinical evidence of mental retardation. Subjects were asked to participate during the first part of their inpatient stay. All patients were on antipsychotic medication at the time of our study. 25% of them were receiving 1st generation antipsychotics (10% were taking oral medication and 90% were receiving long acting therapy) and 75% were receiving 2nd generation antipsychotics (19% of these were receiving clozapine). As for patients with mood disorders with psychotic features a mood-stabilizing medication was administered on top of the antipsychotic medication to all 6 bipolar patients—all of these patients were evaluated during a manic episode (two patients received lithium monotherapy, two received lithium and sodium valproate, one received sodium valproate monotherapy, and one received lithium and lamotrigine). One patient suffering from major depressive disorder with psychotic features was receiving antidepressant medication, specifically mirtazapine. 

The patients had different delusional contents; 80% of the patients had delusions with grandiose, persecutory, and reference contents. Remaining subjects had guilt, religious, or bizarre delusions.

## 3. Methods

First part of the study: 92 subjects were assessed by means of Scale for Assessing Positive Symptoms (SAPS, [17]) and the Scale for Assessing Negative Symptoms (SANS, [18]) and during the same session, the clinical interview for the Theory of Mind test was performed. Subjects and interviewers were well acquainted, thus facilitating open, relaxed conversation. They were reminded that the content of the interview would be used for the study. The patients were asked to tell the interviewer the story of their illness. Each interview began with the question: “I would like to know some more about your story. Please feel free to tell me as much as you remember about your current and past problems, how they began and progressed.” The questions used to assess the delusions were those suggested by Othmer and Othmer, for example, “What is going on?”, “Why is it going on?”, “What kind of thoughts did you have when you were last hospitalised?”, “Where do your thoughts lead?”, “What are you going to do about them?” [19]. The subjects were encouraged to be as detailed as possible when describing their delusions. At the end of the interview, each patient was asked the same question, that is, if he/she considered the content of his/her delusions believable. The questions were “Do you really think that what you just told me is believable? Do you have any doubts about it?” Then the interviewer asked each patient the question, “If you were the interviewer would you consider what you just told me believable? If someone else told you what you just told me, would you believe him/her?” Answers were scored according to the following.

**Question 1**

The patient's opinion about his/her ideas, that is, if he/she considered his/her own delusional ideas believable.

**Question 2**

The patient's idea about the interviewer's point of view, that is, if the interviewer could consider the patient's delusions believable.

Each interview lasted about 1 hour.

Second part of the study: the interviews were tape recorded and transcribed during the first part of the study. Subsequently, (with a delay of 2-3 weeks) 79 out of the 92 subjects, participated in the second part of the study. At this time, 29 of these 79 patients had been discharged. Each patient listened to his/her interview reenacted by actors (the content was identical but the voices were different). At the end of the tape session, the interviewer asked the same questions. The questions were:

1. “Do you think that the interviewed subject presents believable contents?” and
2. “If you were the interviewer would you consider what the other person told him/her believable?”

Answers were scored according to the following.

**Question 3**

The patient's opinion about his/her ideas, that is, he/she considered his/her delusional ideas believable even when presented by another person.

**Question 4**

The patient's idea about the interviewer's point of view, that is, if the interviewer could consider the patient's delusions believable.

## 4. Statistical Analysis

Statistical analysis used Chi-square and ANOVA. ANOVA was performed on age, age at onset, duration of illness, years in school, sex, diagnosis (schizophrenia, psychotic mood disorder, or other psychotic disorder), SANS and SAPS score, item “delusions” score, and improvement of item delusion between first and second clinical interview for the two groups of patients, that is, those who considered believable (do not achieve insight) and not believable (achieve insight) both for the first interview (Questions 1 and 2) and the second interview (Questions 3 and 4).

## 5. Results

After the first interview, 91 out of 92 patients stated that what they said during the interview (their delusion) was believable for them. However, 12 patients thought it was not believable from another person's point of view. After the second interview, 21 of the 79 patients who underwent the second part of our study, stated that the content of the tape was not believable. 17 of these 21 thought it was not believable from the interviewer's point of view whereas 4 of them thought it was not believable both for them and for the interviewer. None of the demographic and clinical variables was correlated with the answers the patients gave at the first interview. However, the answers that the patients provided after the second interview (Table 1) showed a strong correlation with two of the variables, namely, item delusion score at second interview (*P* < .027), and improvement of item delusion score between first and second clinical interview (*P* < .001). Patients with lower item delusion score and a higher improvement of their item delusion score from first to second interview were more likely to provide a negative answer to our questions at the second interview (Question 3, Question 4, or both questions). Obviously, patients could express the same opinion about their delusion at first and second clinical interview, or provide different answers. Most patients (77%) expressed the same opinion whereas 23% expressed a different opinion at the second clinical interview. Analyzing the characteristics of the second group of patients (Table 2), we found that the three of the variables were strongly correlated with the change of opinion, namely, total SAPS score at second interview (*P* < .048), item delusion score at second interview (*P* < .013), and improvement of item delusions score between first and second clinical interview (*P* < .001). Specifically, patients who gained insight (see Table 3) had a lower total SAPS score and item delusion score at second interview, and had a significant improvement of their item delusion score. Vice versa, patients who lost insight had a higher SAPS and item delusions score at second interview, and their item delusion score was higher at second interview than at first interview. Based on responses in the first and the second interview, the patients could be divided into six classes and classified into level of insight (Table 3). 53 patients showed a lack of insight and 7 patients showed partial insight. 14 patients showed either partial or complete gain of insight while 5 patients showed loss of insight.

## 6. Discussion

As for the first part of the study, 91 patients out of 92 state what they said is believable for them. 12 patients out of these 91, however, considered what they said was not believable for the interviewer. This indicates that, similarly to Tegner and Marcels's study, shifting from first- to third-person perspective allows some patients to gain insight (albeit partial). This may indicate that these patients have a functioning ToM and are therefore able to gain insight when asked to modify their cognitive perspective. As mentioned, none of the variables we considered was statistically correlated with the answers the patients gave after the first part of the study. In the second part of the study data are available for 79 patients. After the second interview, 21 patients state what they heard from the tape is not believable; 17 of these patients think it is not believable from the interviewer's point of view, 4 think it is unbelievable both for them and for the interviewer.

Statistical analysis showed a correlation between a negative answer at Questions 3 or 4 (or both) and item delusions score at second interview and improvement at item delusions at second interview. This means patients that were less delusional at second interview gave a negative answer to Questions 3 and 4 or both. These patients also show a significant improvement at item delusion score between the first and the second clinical interview. 

The observation that patients who are less delusional have better insight is consistent with literature data and may depend on the fact that psychotic patients have a rigid cognitive style and a certain overconfidence in their opinions. Also, literature data indicate that the occurrence of delusions is associated with low self-reflectiveness and high self-certainty, possibly reflecting low cognitive insight [20]. Studies comparing psychotic delusional patients with psychotic nondelusional patients show that psychotic delusional patients are overconfident in their opinions whereas psychotic patients who are not delusional have a cognitive style which is similar to healthy controls [21]. 

We also analyzed which patients expressed different opinions about their delusion at the first and at the second clinical interview, and how their opinion changed. Most patients (61), give the same answers at both interviews (53 state the delusion is believable both for them and the interviewer, 7 stating it is believable for them but not for the interviewer, 1 stating it is unbelievable for both). These patients show no significant variation at their item delusions scores between the first and the second clinical interview. 

18 patients expressed different opinions about their delusion at the first and the second interview. In particular, 10 switch from thinking that the delusion is believable both for them and the interviewer to thinking it is unbelievable for the interviewer (gain partial insight); 3 go from thinking the delusion is believable for both to thinking it is unbelievable for both (gain complete insight); 5 patients lose insight stating the delusion is unbelievable for the interviewer after the first interview and stating it is believable both for them and the interviewer after second interview. Patients who gained partial insight improved by a mean of 7.9 points at their item delusion at the second interview. Patients who gained complete insight improved by a mean 12.3 points; patients who lost insight worsened in their item delusion score (on average 1.8 points) but had a mean overall item delusion score at the second interview which was significantly higher than the score of patients who gain partial or complete insight (23.6 points versus 10.9 and 10.3, resp.).

## 7. Conclusions

Our data indicate the improvement of delusions positively correlates with the capacity to gain insight, and the greater the improvement in delusion, the better the insight (a higher improvement was displayed by patients who gained complete insight). Overall, our data indicate that the shift from first to third person allows certain patients to gain insight, by using their ToM (making inferences about the interviewer's point of view). There is also evidence that the patients ability to achieve insight into delusions is strongly related to the intensity of the delusion itself. The less delusional the patients are, the more likely it is that they will have insight into their delusions, regardless of their diagnosis.

## Figures and Tables

**Table 1:** ANOVA for delusion score and improvement of delusion scores at second interview.

		*df*	*F*	*P*-value
Delusion score 2	Between groups	2	3.79	<.027
Improvement of delusion	Between groups	2	11.84	<.000

**Table 2:** ANOVA for change of opinion from 1st to 2nd interview.

		*df*	*F*	*P*-value
SAPS score 2	Between groups	5	2.363	<.048
Delusion score 2	Between groups	5	3.110	<.013
Improvement of delusion	Between groups	5	6.563	<.001

**Table 3:** Patients were divided into classes according to the answers they gave to the questions that they were asked after the first and the second interview. Based on the responses the patients could be classified as either having lack of insight, loss of insight, partial gain of insight, or complete gain of insight.

Class	1st interview	Insight	2nd interview	Insight	Number
1	Believable for me	Lack of insight	Believable for me	Lack of insight	53
	Believable interviewer		Believable interviewer		

2	Believable for me	Partial insight	Believable for me	Partial insight	7
	Unbelievable interviewer		Unbelievable interviewer		

3	Believable for me	Lack of insight	Believable for me	Gain partial insight	10
	Believable interviewer		Unbelievable interviewer		

4	Believable for me	Lack of insight	Unbelievable for me	Gain complete insight	3
	Believable interviewer		Unbelievable interviewer		

5	Unbelievable for me	Complete insight	Unbelievable for me	Complete insight	1
	Unbelievable interviewer		Unbelievable interviewer		

6	Believable for me	Partial insight	Believable for me	Lose insight	5
	Unbelievable interviewer		Believable interviewer		
